# Androgen receptor-beta mRNA levels in different tissues in breeding and post-breeding male and female sticklebacks, *Gasterosteus aculeatus*

**DOI:** 10.1186/1477-7827-10-23

**Published:** 2012-03-28

**Authors:** Erik Hoffmann, Anders Walstad, Johnny Karlsson, Per-Erik Olsson, Bertil Borg

**Affiliations:** 1 Department of Zoology, Stockholm University, S-106 91 Stockholm, Sweden; 2 School of Science and Technology, Örebro Life Science Center, Örebro University, SE-701 82 Örebro, Sweden

## Abstract

**Background:**

Androgens induce male characters by activating androgen receptors (AR). Previous quantitative studies on AR in fishes have been limited to few tissues and/or a single season/reproductive state. The aim of this investigation was to study the possible role of AR-beta expression levels in the control of male traits in the three-spined stickleback. To that end, AR-beta expression levels in major tissues in breeding and post-breeding male and female sticklebacks were examined.

**Methods:**

AR-beta mRNA levels were quantified in ten tissues; eye, liver, axial muscle, heart, brain, intestine, ovary, testis, kidney and pectoral muscle in six breeding and post-breeding males and females using reverse transcription quantitative PCR.

**Results:**

Breeding in contrast to post-breeding males built nests and showed secondary sexual characters (e.g. kidney hypertrophy) and elevated androgen levels. Post-breeding females had lower ovarian weights and testosterone levels than breeding females. AR-beta was expressed in all studied tissues in both sexes and reproductive states with the highest expression in the gonads and in the kidneys. The kidney is an androgen target organ in sticklebacks, from which breeding males produce the protein spiggin, which is used in nest-building. There was also high AR-beta expression in the intestine, an organ that appears to take over hyperosmo-regulation in fresh water when the kidney hypertrophies in mature males and largely loses this function. The only tissue that showed effects of sex or reproductive state on AR-beta mRNA levels was the kidneys, where post-breeding males displayed higher AR-beta mRNA levels than breeding males.

**Conclusion:**

The results indicate that changes in AR-beta mRNA levels play no or little role in changes in androgen dependent traits in the male stickleback.

## Background

Androgens stimulate masculine traits such as development of male secondary sexual characters, male reproductive behaviour and spermatogenesis via interactions with nuclear androgen receptors (AR). Among teleost fishes, complete AR cDNAs have been cloned in e.g. Japanese eel, *Anguilla japonica *[1], rainbow trout, *Oncorhynchus mykiss *[2] and red seabream, *Pagrus major *[3].

In some teleost fishes, two different AR genes have been cloned e.g. Japanese eel [1], Burton's mouthbrooder, *Astatotilapia burtoni *[4] and Western mosquitofish, *Gambusia affinis *[5]. The nomenclatur used for AR subdivision into alpha and beta is based on the first report of two forms present in Japanese eel [6]. We base the nomenclature in the present study on available phylogenetic analyses [6,7], to name the different AR forms alpha or beta in accordance with their relationship to the Japanese eel forms.

Recently, an AR-beta with two splicing variants was cloned from the kidney of the three-spined stickleback *Gasterosteus aculeatus *[7]. In this species the kidney is an androgen target organ, which hypertrophies in the breeding male and produces glue used in nest-building. This stickleback AR-beta showed high affinity binding (K_d _in the nM range) for androgens and low number of binding sites (B_max _in the pmol/g tissue range), which is in consistent with nuclear receptors [7]. Furthermore, the trans-activation function of stickleback AR was verified in transfected human and zebra fish liver epithelial cell lines upon stimulation by androgen, preferentially by 11-ketotestosterone (11KT).

Recently, a second stickleback AR has been reported [8]. Based upon amino acid sequence analysis this AR belonged to the alpha group of teleost AR, though referred to in that article as AR-B. It is so far not known whether this is a functional receptor in the stickleback.

Expression of AR transcripts has been studied in several fishes with different techniques e.g. Northern blot, semi-quantitative reverse transcriptase PCR and real-time PCR. However, most studies have been limited to a few tissues, the male sex (sometimes with the ovary added), and/or qualitative or semi-quantitative techniques. AR-beta mRNA was measured in eye, liver, brain, gonads, kidneys, skin and muscle in adult male and female zebrafish [9] and in liver, brain, gonads and pituitary of male and female *Spinibarbus denticulatus *[10] at different stages of maturation.

In order to obtain a more complete picture of the roles of AR in fishes, expression profiles need to be obtained from more organs and under different reproductive conditions. To that end, 15 tissues/organs were sampled from breeding and post-breeding male and female sticklebacks and AR-beta mRNA levels were measured using reverse transcription quantitative PCR (rt-qPCR).

## Methods

### Animals

Handling of the fish and experiment design were approved by the Northern Stockholm Animal Research Ethical Committee.

Adult non-breeding three-spined sticklebacks, *Gasterosteus aculeatus *were caught in the Öresund, in the south of Sweden. The fish were kept in aquaria with filtered and aerated brackish water (0.5% salinity), under short photoperiod (8 h light: 16 h darkness), at 5-8°C to maintain them in non-breeding "winter" condition. The bottom was covered with sand and there were tubes of ceramic material that provided hiding places. The fish were fed daily with frozen red midge larvae or mysids.

### Experimental protocol

In order to induce maturation, groups of fish were transferred to long photoperiod (LD 16:8) and 20°C, otherwise as above. Only fish that matured, i.e. females that developed roe-swollen bellies and males that developed red breeding colors were used. The breeding fish were transferred to individual 50-liter aquaria containing sand, filamentous algae and filtered water. All males in this group built nests and all females had ovulated at least once. The breeding fish were sampled after one and a half months in LD 16:8 and 20°C. The post-breeding fish were sampled after three months in LD 16:8 and 20°C, when the fish had entered the refractory phase, i.e. the males had lost their breeding coloration and the females showed no swollen bellies.

The fish were anaesthetized with 0.1% 2-phenoxyethanol (Sigma, St. Louis, MO, USA) and weighed. Blood samples for steroid measurement were collected from the severed caudal peduncle and stored at - 70°C until processed.

Part or whole organs/tissues; i.e. eye, brain (pituitary included), spleen, gills (filaments separated from the gill arches), heart, liver, a c. 1 cm long posterior segment of the intestine, trunk kidneys (referred to as "kidney"), head kidney, right pectoral muscle (both inner and outer musculature), axial muscle (sampled from the back beneath the third back-spine), pectoral and caudal fins, testes and ovary were snap frozen and stored at -70°C. Intestines were rinsed with sterile 0.9% NaCl before freezing to reduce numbers of bacteria. Kidneys and gonads were weighed prior to freezing in order to calculate kidney somatic index (KSI, kidney weight/body weight × 100) and gonad somatic index (GSI, gonad weight/body weight × 100).

### Steroid measurement

Testosterone (T) and 11KT levels were assessed by radioimmunoassay according to [11]. In brief, individual plasma samples were diluted to 300 μl in RIA buffer, heated at 80°C for 60 min., centrifuged at 13000 rpm for 15 min. after which the supernatant was extracted and stored at 4°C until being assayed. To incubation vials following was added; 50 μl aliquots of the sample, 50 μl RIA buffer, 50 μl of the radiolabelled steroid (3H-11KT, a generous gift from Dr Alexander Scott, Cefas, UK or 3H-T, Amersham International) 30-35 × 10^3 ^dpm/50 μl and 200 μl of steroid antiserum (11KT or T antisera were generous gifts from Dr Helge Tveiten, University of Tromsø, Tromsø, Norway). All samples were run in duplicate. Vials were vortexed and incubated over night at 4°C. Free, unbound steroid was separated from bound steroid with dextran charcoal suspension (Activated charcoal and Dextran T-70). Following 5 min centrifugation at 3900 rpm, the supernatant was poured into scillation vials containing 4 ml scintillation fluid (OptiPhase Hi Safe II, LKB Wallac) and run in the counter (1214 Rackbeta liquid scintillation counter, LKB Wallac). The detection limit for the assay was ca. 2 ng/ml, and intra- and interassay coefficients of variance were 5.4 and 7.0%, respectively.

### RNA extraction, cDNA synthesis and real time quantitative PCR

Tissue samples from six breeding and post-breeding males and females were homogenized and total RNA was extracted using TRI reagent (Sigma-Aldrich). Total RNA was treated with DNase Turbo (Ambion Inc., Applied Biosystems) and quantified using RiboGreen RNA reagent (Molecular Probes, Invitrogen) and the GENios Pro (TECAN).

First-strand cDNA synthesis was performed on 100 ng of total RNA using 50 μM random decamer primers **(**Ambion Inc., Applied Biosystems), 200 units of MMLV-RT (Invitrogen) and 40 units of RNase Inhibitor (Invitrogen) incubated at 25°C for 10 min, followed by 37°C for 50 min and 70°C for 15 min.

A standard for AR was prepared from sense AR cRNA generated from the three-spined stickleback AR-beta cDNA clone (Gene Bank Accession no AY24706) using MEGAscript T7 kit (Ambion Inc., Applied Biosystems). A serial dilution of AR cRNA was reverse transcribed to cDNA as described above and used as a standard in the quantitative PCR (qPCR) assay.

Gene-specific primers for AR (Forward) (5'-CACAAATGGTCTTCCTCAACATCCT-3'; (Reverse) 5'-CGTGCCCTGCGTTCAC-3') and probe (5'-CACCTCGGGTTCAATG-3') based on the stickleback AR-beta sequence (Gene Bank Accession no AY24706) were designed by TaqMan (Applied Biosystems, Warrington, UK). Sequence analysis of the primer and probe sequences against the newly reported stickleback AR-alpha cDNA (ENSEMBL Transcript ID ENSGACT00000024538) [8], confirmed that the sequences did not match the new AR sequence. The specificity of AR primers were tested by PCR and resulted in a single band of expected length (68 bp) as determined by agarose-gel electrophoresis. For reference gene, we used a pre-developed 18S ribosomal RNA assay by TaqMan, (Applied Biosystems, Foster City, CA). A serial dilution of sample cDNA was used as a standard for 18S rRNA.

Reverse transcription qPCR was performed on ABI Prism 7000 Sequence Detection System (PE Applied Biosystems, Foster City, CA). All samples were run in duplicate in a final volume of 50 μl, containing 25 μl TaqMan Universal PCR mastermix, 2.5 μl primer (18 μM), probe (5 μM), 1 μl cDNA and 21.5 μl nuclease free water (Ambion Inc., Applied Biosystems). Following amplification conditions were applied: at 50°C for 2 min., 95°C for 10 min. followed by 40 cycles of 95°C for 15 sec, 60°C for 1 min.

AR and 18S rRNA standards and negative controls (included all reagents except for template) were included in each PCR run. Calibrator samples for AR and 18S rRNA were included in each PCR run, to calculate the inter-assay variation. The inter-assay variation was included in the calculation of sample cycle threshold (C_T_). The amount of AR and 18S rRNA in each sample was determined according to Standard Curve Method (Applied Biosystems User Bulletin #2, 2001) by comparing mean sample C_T _values to the standards. PCR efficiency analysis was performed on each reaction.

Serial dilutions of AR and 18S rRNA standards produced linear plots of input cDNA vs C_T _with r^2 ^> 0.99. AR and 18S rRNA standards showed no differences in slope or intercept between different PCR runs (Linear regression analysis, p = 0.71 and p = 0.11 and p = 0.43 and p = 0.23, for AR and 18S rRNA respectively) except for PCR runs involving head kidneys, pectoral and caudal fins, where standards showed higher intercepts. Therefore, head kidney, pectoral and caudal fins are not comparable with the other organs. The intra- and interassay coefficient of variation for *ar *mRNA levels were 9.9% and 17.8%, respectively.

### Statistical analysis

Statistical analyses were performed using Prism version 3.0 (GraphPad, Software, San Diego, CA, USA). All data were tested for normality and homogeneity of variance using Kolmogorov-Smirnov and Bartlett's test, respectively. KSI and GSI values were arcsine square root transformed, plasma androgen levels were log transformed and AR/18S rRNA values were (-1 * log (y)) transformed, prior to analysis to meet assumptions of homogeneity of variance. Statistical differences in KSI and 11KT and T plasma levels and 18S rRNA expression between experimental groups were tested using one-way ANOVA followed by the Tukey's post-hoc test. Differences in GSI between breeding and post-breeding females and males respectively were assessed by ANCOVA. Differences in slope and intercept for AR and 18S rRNA standards, respectively, in different PCR runs were tested in Linear regression analysis. Two-way ANOVA followed by Bonferroni post-hoc test was used to examine differences in tissue AR-beta mRNA levels with sex and reproductive state as factors. One-way ANOVA followed by the Tukey's post-hoc test was used to determine relative AR-beta mRNA levels in different tissues. In this comparison all samples from each tissue regardless of sex and state of maturity were pooled. This was considered justified since there was only one case with significant effects of sex and maturity within organs (see below). Results were considered statistically significant at p < 0.05. All data are presented as mean ± S.E.

## Results

Breeding females displayed higher body weight compared to the other groups (Table 1). All breeding males built a nest and displayed breeding coloration and hypertrophied kidneys which none of the post-breeding males did. Breeding males had higher KSI than the other groups (Tukey's post-hoc test, p < 0.001 in each of six comparisons), but there were no differences between the latter (Table 1). GSI was higher in breeding than in post-breeding females (ANCOVA, p < 0.001) and males (p < 0.05) (Table 1). Both 11KT and T levels were higher in breeding males compared to the other groups (Tukey's post-hoc test, p < 0.001 in each of the six comparisons) and breeding females showed higher T levels than post-breeding males and females (p < 0.05) (Table 1).

**Table 1:** Experimental animals

	Breeding		Post-breeding	
	**Males**	**Females**	**Males**	**Females**

**Body weight**	1.71 ± 0.15	2.65 ± 0.34	1.89 ± 0.14	2.28 ± 0.13

**KSI**	3.34 ± 0.12 ^a^	0.49 ± 0.07	0.82 ± 0.04	0.58 ± 0.05

**GSI**	0.52 ± 0.07 ^b^	17.80 ± 2.38 ^c^	0.27 ± 0.02	4.16 ± 0.65

**11KT**	337 ± 55 ^a^	6.9 ± 1.8	6.1 ± 1.9	4.0 ± 0.5

**T**	78.1 ± 17.0 ^a^	19.4 ± 8.3 ^d^	4.5 ± 0.7	5.1 ± 0.6

Body weight (g), kidney somatic index (KSI, kidney weight/body weight × 100), gonad somatic index (GSI, gonad weight/body weight × 100) and plasma levels (ng/ml) of 11-ketotestosterone (11KT) and testosterone (T) in breeding and post-breeding male and female sticklebacks. Means ± S.E. are shown. n = 6. ^a ^(Tukey's post hoc test, < 0.001) compared to all other groups. ^b ^(ANCOVA, p < 0.05) compared to post-breeding males. ^c ^(ANCOVA, p < 0.001) compared to post-breeding females. ^d ^(Tukey's post-hoc test, p < 0.05) compared to post-breeding males and females.

### Expression of AR and 18S rRNA

**Figure 1:**
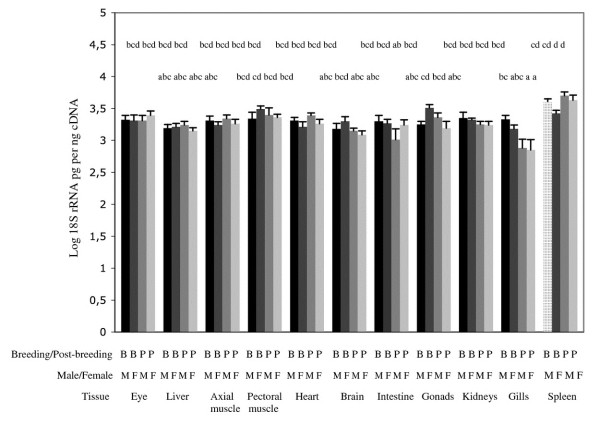
**18S rRNA expression levels in tissues in breeding (B) and post-breeding (P) male and female sticklebacks**. Mean ± S.E. is shown. n = 6. Groups, which do not share at least one letter, are significantly different p < 0.05. One-way ANOVA followed by Tukey's post-hoc test.

There was a difference in the 18S rRNA levels per ng cDNA in different tissues (Figure 1) (one-way ANOVA, p < 0.001). The highest levels of 18S per ng cDNA were found in the spleen and the lowest in the gills.

The relative 18S levels in spleens and gills were often significantly higher and lower respectively, than in many other organs (see Figure 1 for details). For this reason, the AR-beta levels in these organs, which appeared to be low, are not shown.

Among the other organs only two comparisons were significantly different; the intestine from post-breeding males had less 18S rRNA than pectoral muscle and ovary in breeding females (both p < 0.05), for this reason the AR-beta levels in the former (which appeared to be similar to the other intestine samples) is not shown.

**Figure 2:**
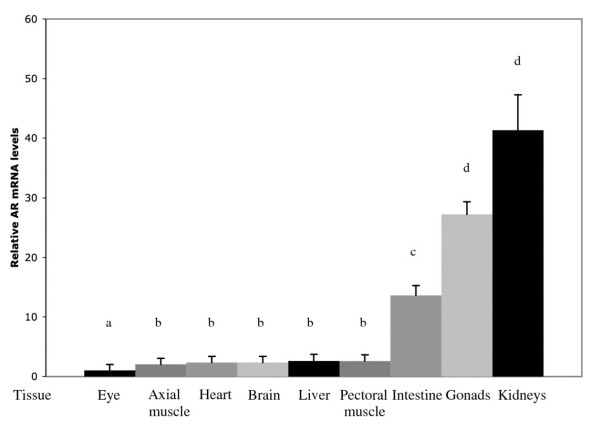
**Relative AR mRNA levels in tissues in male and female sticklebacks**. Mean ± S.E. is shown. n = 24 for all tissues except for the intestine, n = 18. Groups, which do not share at least one letter, are significantly different p < 0.05. One-way ANOVA followed by Tukey's post-hoc test.

AR-beta was expressed in all tested tissues with the highest AR-beta mRNA levels in the kidneys and gonads while the lowest levels were observed in the eyes (Figure 2). The AR-beta mRNA levels were different in different types of tissues (one-way ANOVA, p < 0.001). Axial muscle, heart, brain, liver and pectoral muscle had higher AR-beta mRNA levels than the eye, (in each comparison, p < 0.05). Values for head kidney, caudal and pectoral fins are not comparable with the rest (see above) and are not shown in the figure.

The highest AR-beta mRNA levels were found in kidneys and gonads, which were significantly higher than those in all other tissues (Tukey's test p < 0.001 in each test, except for the comparison between the intestine and gonads, p < 0.05). The level in the intestine was significantly higher than in all other tissues except kidneys and gonads (p < 0.001).

**Figure 3:**
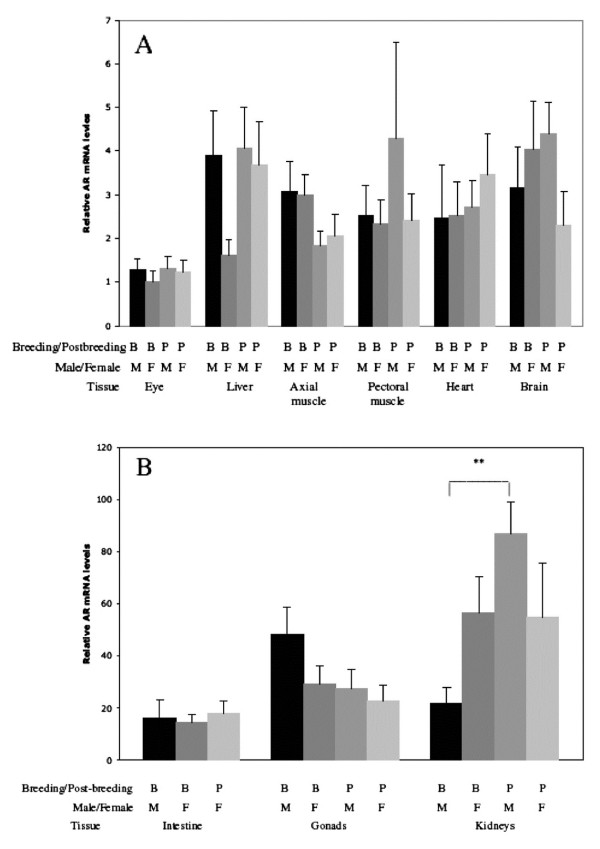
**Relative androgen receptor expression levels in tissues in breeding (B) and post-breeding (P) male and female sticklebacks**. Note different scales in Figure 3A and B. Mean ± S.E. is shown. n = 6. For statistic analysis see results. ** p < 0.01.

There was no effect of sex or reproductive state on the AR-beta mRNA levels in brain, axial musculature, eye, pectoral muscle, heart, gonads and liver (Figure 3A and 3B). There was, however, a significant effect of reproductive state on renal AR-beta mRNA levels (two-way ANOVA, p = 0.017) (Figure 3B). Post-breeding males displayed higher AR-beta mRNA levels in the kidneys compared to breeding males (Bonferroni post-hoc test, p < 0.01) but there were no significant differences in AR-beta mRNA levels in the other comparisons. No effect of sex or interactions between sex and reproductive state on AR-beta mRNA levels in the kidneys were found.

## Discussion

AR-beta was expressed in all tissues in both sexes and reproductive states. The ubiquitous AR-beta expression in adult stickleback tissues is largely consistent with a number of earlier studies in teleosts (e.g. [2,5,6,9,10].

The AR-beta mRNA levels in stickleback tissues range up to two orders of magnitude with the highest levels in the kidneys and the gonads. The kidney is a well-known androgen target tissue in sticklebacks. The male kidney hypertrophies in the breeding season and produces a glue-protein, spiggin, which is used in the building of the nest [12]. Kidney hypertrophy/spiggin production is stimulated by androgens, especially 11-ketoandrogens, and is suppressed by castration [12-15]. The androgens act on the kidney level. Androgen treatments induce transformation of kidney secondary proximal tubule cells into glandular cells in vitro [16]. Androgens induce spiggin synthesis in kidney cell [17] and tissue culture [18]. Many differences in the structure of renal corpuscles in mature compared to immature males have been observed using electron microscopy [19]. Kidney tissue cultures treated with T or 11KT also showed some of these effects, i.e. activated mesangial cells and podocytes [20]. Androgens can stimulate the stickleback kidney also outside the breeding season [20] and in females, where otherwise kidney hypertrophy and spiggin levels are low [21,22].

Similar to sticklebacks, the AR expression was higher in the kidneys than in many other studied tissues in zebrafish [9], male half-smooth tongue sole (*Cynoglossus semilaevis*) [23] and rice field eel (*Monopterus albus*) [24]. For these and most fishes, no sex dimorphism in the kidney is known and the possible role of androgens has not been studied.

High AR mRNA levels in the testes have also been found in several previous studies on fishes (e.g. zebrafish (AR-beta) [9], *Spinibarbus denticulatus *(AR-beta) [10], sea bass (AR-beta) [25], three spot wrasse (*Halichoeres trimaculatus*) (AR-beta) [26]). This is not surprising since androgens are necessary for spermatogenesis in most studied vertebrates, including teleosts. A stimulating in vitro effect on spermatogenesis by androgens, preferentially by 11KT, has been shown in the Japanese eel [27,28]. In contrast to most other studied teleosts, androgens inhibit spermatogenesis in stickleback [29,30]. Males display secondary sexual characters and high levels of 11KT and T during breeding season when spermatogenesis is quiescent [31]. Spermatogenesis commences at the end of the breeding season, when circulating androgens levels are very low [32]. Administration of androgens, particularly 11-ketoandrostenedione (11-KA, which is converted extra-testicularily to 11KT [31]) at the end of breeding prevents the onset of spermatogenesis [29,32].

In the stickleback, AR-beta mRNA levels were as high in the ovaries as in the testes. Similar findings were reported in the three spot wrasse [26]. High mRNA levels of AR-alpha, but not of AR-beta were found in ovaries of rainbow trout [2]. In zebrafish [9] and sea bass [25] there was higher level of AR-beta mRNA in the testes than in the ovary. Ovarian AR-beta mRNA levels were at most 1% of those found in testes in *Spinibarbus denticulatus *[10]. It is not known what biological role(s) AR expression may have in the stickleback ovary or in female fish in general. Female teleost fishes, including the stickleback [33], display much lower levels than males of 11-KT, the most effective androgen in fishes [33]. However, females have equal or even higher circulating levels of T than males in most studied teleosts [33], including breeding sticklebacks sampled in the field [34]. Testosterone may have action(s) by itself or after conversion to more effective androgens. However, it may also be converted to estrogens. Indications for androgenic actions on the ovary are provided by effects of the anti-androgen flutamide on fathead minnows, *Pimephales promelas *[35]. High doses of flutamide reduced the number of spawnings and eggs/spawning. Treated females had higher plasma levels of vitellogenin and T but not of 17β-estradiol, less oocyte maturation and more atretic follicles than control fish [35]. The low maturation and high levels of vitellogenin may suggest an impaired uptake of vitellogenin into the eggs.

AR-beta mRNA levels were also high in the stickleback intestine. The intestine has been proposed to take over the kidneys' role as a freshwater osmoregulatory organ in mature stickleback males [36]. The androgen-induced transformation of the kidneys into a glandular organ excreting spiggin also leads to a reduced ability to re-uptake ions and to excrete surplus of water [36]. On the other hand a higher intestinal fluid secretion was found in mature than in immature stickleback males [36]. Furthermore, the basal labyrinth, an intracellular membrane system characteristic for ion and water transporting epithelial cells, was more developed in the enterocytes in mature males and androgen treated females than in immature males and females [36]. The high AR-beta expression in the intestine suggests that androgen effects are likely to be exerted directly on the intestine level. A role of the intestine in hyperosmoregulation is not known from other fishes. On the contrary, fish in seawater drink to compensate osmotic water losses and the water is absorbed by the intestine [37]. In salmonids, sexual maturation and androgen treatments impair the hypoosmoregulatory ability [38], but it is not known whether this response involves the intestine. Expression of AR in the intestine has only been found in a few teleost fishes. AR expression was found in all investigated tissues, including the intestine in male half-smooth tongue sole [23].

AR-beta was present in the stickleback brain, but the levels were low. Androgens stimulate reproductive behavior in the male stickleback [39], but the effects may be exerted via a small proportion of the brain cells. AR(s) were also expressed in brains of several other fishes [2,4,5,9,25,26,40].

The AR-beta mRNA level in the stickleback liver was low. This is in general agreement with findings in the zebrafish [9]. However, in *Spinibarbus denticulatus *[10], Mozambique tilapia [40] and rice field eel [24] liver AR mRNA levels were higher than in any other studied organs. Yolk proteins like vitellogenin are produced by the liver under stimulation of estradiol. High AR expression in the liver may suggest that also androgens are involved in the control of vitellogenesis. This is also supported by in vitro effects of androgen on the liver of Japanese eel [41]. In this species, AR-alpha was found in the liver, whereas AR-beta was not [6].

In the stickleback, both pectoral and axial muscle contained low levels of AR-beta mRNA. AR(s) in musculature have also been found in some other fishes [6,9,10]. Male sticklebacks have larger pectoral muscle than females and use their pectoral fins vigorously when ventilating the nest [42]. Castration reduced the weight of pectoral muscles but T or 11KT treatment did not restore it [42].

There was no significant effect of sex on AR-beta mRNA levels in any studied organ in the present study. There are very few other studies where AR mRNA has been studied quantitatively in both sexes of fishes. In zebrafish there were about twice as much AR-beta mRNA in testes as in ovaries and in male than in female muscle, whereas there was no significant effect of sex in brain, kidney, liver, skin and eye [9]. AR-beta mRNA levels were measured in pituitary, brain, gonads and liver in male and female *Spinibarbus denticulatus *[10]. The levels were clearly higher in testes than in ovaries, but there were lower levels in the pituitary of males than in females.

We found no significant differences in AR-beta mRNA levels in the kidneys between breeding male and female sticklebacks whereas in another study a 1.4-fold higher kidney AR-beta expression in mature males than in mature female sticklebacks was reported [14].

Although breeding and post-breeding fish, particularly in males, showed large differences in 11-KT and T plasma levels, there were no differences in AR-beta mRNA levels in most studied tissues, except for the kidneys. Unexpectedly, we found higher AR-beta mRNA levels in the kidneys of post-breeding compared to breeding males. Breeding males in the present study displayed well-developed hypertrophied kidneys and high T and 11KT levels. Conversely, none of the post-breeding males showed hypertrophied kidneys and their androgen levels were very low, indicating that they had left the reproductive state. High AR-beta mRNA levels in the kidneys of breeding males are likely necessary for the high spiggin synthesis. What function(s), if any, males may have of continuous high AR-beta expression in the kidneys shortly after the reproductive period is unknown.

These results suggest that androgens have a little, if any, effect on AR-beta expression in most organs and at least no positive effect in the kidney. No support for auto-regulation of AR-beta mRNA or protein levels (binding capacity) in stickleback kidneys was found when intact females and castrated males were treated with androgens i.e. T, 11KA or 5-alpha dihydrotestosterone [7]. Furthermore, no effect of methyltestosterone on kidney AR-beta expression was found in intact female sticklebacks, although this treatment increased spiggin synthesis by five orders of magnitude [14].

## Conclusion

Here we demonstrated that AR-beta is expressed in all major tissues in adult three-spined sticklebacks. Most tissues exhibited low AR-beta mRNA levels but in the known androgen target organs i.e. the testes and kidneys, and in the ovary and the intestine, the AR-beta mRNA levels were considerably higher, up to 100-fold. There were no clear effects of sex and reproductive states in AR-beta mRNA levels except for the kidneys, where post-breeding males displayed higher AR-beta mRNA levels than breeding males.

Despite large differences in androgen levels between breeding and post-breeding fish, most investigated tissues exhibited similar AR-beta mRNA levels. These results suggest that changes in androgen target tissues during the reproductive cycle are controlled by changes in androgen levels and not by changes in AR-beta mRNA levels.

## Abbreviations

T: Testosterone; 11KT: 11-ketotestosterone; AR: Androgen receptor.

## Competing interests

The authors declare that they have no competing interests.

## Authors' contributions

EH, BB, JK and P-EO designed the experiments and prepared the manuscript. EH and BB participated in the preparation of the animals and tissues. EH and AW performed RNA extraction, cDNA synthesis. EH performed the RIA, the real time quantitative PCR and the analysis of data. All authors have read and approved the final manuscript.
